# Separase Control and Cohesin Cleavage in Oocytes: Should I Stay or Should I Go?

**DOI:** 10.3390/cells11213399

**Published:** 2022-10-27

**Authors:** Katja Wassmann

**Affiliations:** Institut Jacques Monod, Université Paris Cité, CNRS, 75013 Paris, France; katja.wassmann@ijm.fr

**Keywords:** separase, oocytes, meiosis, cohesin, Rec8, cohesin protection, securin, cyclin B1, Sgo2, aneuploidy

## Abstract

The key to gametogenesis is the proper execution of a specialized form of cell division named meiosis. Prior to the meiotic divisions, the recombination of maternal and paternal chromosomes creates new genetic combinations necessary for fitness and adaptation to an ever-changing environment. Two rounds of chromosome segregation -meiosis I and II- have to take place without intermediate S-phase and lead to the creation of haploid gametes harboring only half of the genetic material. Importantly, the segregation patterns of the two divisions are fundamentally different and require adaptation of the mitotic cell cycle machinery to the specificities of meiosis. Separase, the enzyme that cleaves Rec8, a subunit of the cohesin complex constituting the physical connection between sister chromatids, has to be activated twice: once in meiosis I and immediately afterwards, in meiosis II. Rec8 is cleaved on chromosome arms in meiosis I and in the centromere region in meiosis II. This step-wise cohesin removal is essential to generate gametes of the correct ploidy and thus, embryo viability. Hence, separase control and Rec8 cleavage must be perfectly controlled in time and space. Focusing on mammalian oocytes, this review lays out what we know and what we still ignore about this fascinating mechanism.

## 1. Introduction

Sexually reproducing organisms have to generate haploid gametes harboring half the chromosome count. Fusion of the male and female gamete then re-establishes the original chromosome number and gives rise to the diploid zygote, the first cell of the future embryo. To generate haploid gametes, germ cells have to pass through two specialized cell divisions named meiosis I and II. In the first division, which is reductional, chromosomes consisting of two sister chromatids each, are segregated, whereas in the second meiotic division, sister chromatids -such as in mitosis- are segregated into two daughter cells. Because there is no DNA synthesis occurring between the two divisions, the end product of meiosis is four haploid daughter cells, each containing one copy of the genome [1].

**Figure 1:**
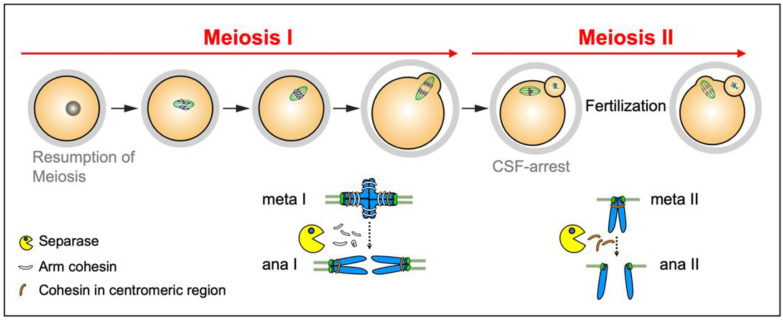
Mouse oocyte meiosis. Prophase I arrested oocytes are found in the ovaries of adult mice. Upon hormonal stimulation, they resume meiosis, with nuclear envelope breakdown and progression through meiosis I, followed by meiosis II. Oocytes arrest in the metaphase of meiosis II (CSF-arrest) to await fertilization, which induces anaphase II and exit from meiosis II. The meiotic divisions in oocytes are highly asymmetric to generate a small polar body that degenerates to maintain the volume of the oocyte for future embryo development. The segregation patterns are fundamentally different in meiosis I and II and are indicated in the scheme below, placed beyond the corresponding oocyte cell cycle stages. Separase (in yellow) removes arm cohesin (white) in meiosis I and centromeric cohesin (orange) in meiosis II. The typical cross-shaped form of paired chromosomes in meiosis I is due to chiasmata holding chromosomes of different parental origins together and the fact that mouse chromosomes are telocentric (centromeres are localized towards the ends of chromosomes).

Female meiosis produces the egg, which upon fertilization, gives rise to the zygote (Figure 1). Meiotic divisions in oocytes are very asymmetric to ensure that the egg maintains all the maternal proteins required for successful embryo development. Unlike male meiosis, oocytes have to observe two cell cycle arrests, one for growth in prophase of meiosis I and a second after so-named meiotic maturation, in meiosis II to await fertilization, and this arrest is called Cytostatic Factor or CSF-arrest [2,3]. Segregation of the genetic material thus has to be perfectly coordinated with the developmental stage of the oocyte.

The aim of meiosis is the generation of new genetic combinations in the offspring. This requires the recombination of stretches of homologous DNA from different parental origins in germ cells before starting the meiotic divisions. Germ cells undergo premeiotic S-phase, whereby the two copies of the genome (one of paternal, one of maternal origin) constituting a diploid organism are duplicated. Proteins of the cohesin complex, which is holding the replicated sister chromatids together, are loaded. This is followed by the pairing of homologous chromosomes at the start of meiosis. A structure named the synaptonemal complex forms and maintains the two chromosomes together, providing the required stability for supporting double-strand breaks and repair. Recombination has to take place between sister chromatids of distinct parental origin, i.e., between the two chromosomes. This is achieved through the so-called homolog bias, which suppresses recombination events between sister chromatids of the same chromosome and, thus, the same parental origin. As excellent reviews on meiotic recombination have been published (for example [4,5,6]), and the scope of this review is meiotic divisions, the reader is thus referred to the existing literature for those aspects of meiosis prior to the divisions.

The different steps of meiotic recombination take place before entry into the first meiotic division. At the transition from prophase I into prometaphase I, all double-strand breaks have been repaired. Some sites of recombination that are defined as crossovers are visible as chiasmata. These recombination events constitute physical connections holding the homologous chromosomes together. Chiasmata are thus extremely important for the correct segregation of chromosomes in meiosis I because they allow the establishment of tension-bearing attachments of chromosomes to the opposite poles of the bipolar spindle. Without them, the two chromosomes segregate at random, or alternatively, chromosomes may precociously separate into two sister chromatids in meiosis I [1,7].

Sister chromatids are held together by cohesin complexes, forming a ring-like structure around the two DNA strands constituting the chromatids. Because of the exchange of genetic information between sister chromatids of the two chromosomes prior to the first meiotic division, cohesin complexes furthermore provide the structure to stabilize chiasmata [8,9]. Segregation of chromosomes in meiosis I and sister chromatids in meiosis II depends on step-wise cohesin removal. Cohesin is removed from chromosome arms in meiosis I and from the pericentromere in meiosis II by the same enzyme, called separase. Maintenance of cohesin in the centromere region is a condition for the proper bipolar attachment of sister chromatids in meiosis II [10]. Without pericentromeric cohesin, sister chromatids are segregated at random because they cannot establish tension-bearing attachments to the bipolar spindle. However, only a very small fraction of centromere-localized cohesin at the pericentromere, is not cleaved in meiosis I in mouse oocytes [11]. How exactly this small fraction of cohesin is protected from cleavage in meiosis I but not meiosis II is still not entirely clear. What is known is that cohesin protection in meiosis I depends on Sgo2-dependent localization of the phosphatase PP2A-B56, which is thought to keep a small fraction of Rec8 in the centromere region dephosphorylated and hence, not cleavable by separase [12,13,14]. However, Sgo2, as well as PP2A-B56, are localized to the pericentromere and co-localize with Rec8 also in meiosis II [15], when Rec8 has to be cleaved, indicating that the protection-deprotection mechanism is more complex than just localization of PP2A-B56 in the vicinity of Rec8 to protect it.

## 2. Separase

The cohesin complex consists of two Structural maintenance proteins (SMCs) and the kleisin subunit. The cohesin complex is removed through cleavage of the kleisin subunit. Separase is a cysteine protease whose activity is required in metaphase in both mitosis and meiosis, where it targets the kleisin Scc1 or Rec8, respectively [16]. It recognizes a conserved cleavage site consensus motif in its substrates, and cleavage is enhanced by a substrate motif close to the cleavage site [17]. Importantly, in mitosis, separase cleavage is required to eliminate cohesin in the centromere region only because arm cohesin is removed in the prophase of mitosis by an alternative pathway, the so-named prophase pathway [18]. The prophase pathway depends on the kinases Aurora B and Cdk1, which counteract Sororin, an inhibitor of the cohesin-release factor Wapl [19,20,21,22,23]. Pericentromeric cohesin in mitosis is protected by Sgo1-PP2A-B56 from prophase pathway-dependent removal. Like this, most cohesin is already removed before metaphase and sister chromatids are held together only in the centromere region. Activation of separase then leads to cleavage of the remaining Scc1 and synchronized separation of the individual sister chromatids into two daughter cells.

In meiosis, Rec8 substitutes for Scc1. To bring about step-wise cohesin removal, Rec8 cleavage is precisely regulated in space and time; however, only separase is required to remove both arm- and centromere-localized cohesin [24]. Accessibility of Rec8, but also tight separase control are hence crucial for correct segregation of the genetic material in both divisions. Below I will first give an overview of separase control in mitosis, followed by a description of our current state of knowledge in oocyte meiosis.

### 2.1. Separase Control in Mitosis

Separase is a “dangerous” enzyme: too early activation can be expected to lead to precocious cleavage of cohesin, hence removing the links necessary to keep sister chromatids paired until they are correctly attached to the bipolar spindle before being separated. If sister chromatids get separated before being correctly attached, they are going to missegregate during anaphase, thus leading to aneuploidies in the resulting daughter cells. Therefore, it is not surprising that several inhibitory mechanisms keep separase under check until anaphase onset.

#### 2.1.1. Securin

In vivo, the main inhibitor of separase is thought to be securin, with around 60% of separase being associated with securin in metaphase-arrested cells [25]. Securin binding to separase inhibits its cleavage activity by functioning as a pseudo-substrate [26,27,28]. Interestingly, securin associates with separase as it has been translated, with securin also being required for the correct folding of separase. Hence, securin not only inhibits separase but also ensures its activity [29].

Securin is a substrate of the APC/C (anaphase promoting complex/cyclosome), an E3-ubiquitin ligase which, in association with its activators Cdc20 or Cdh1, targets key cell cycle regulatory proteins for degradation [30]. Activation of APC/C^Cdc20^ at the metaphase-to-anaphase transition depends on the satisfaction of a checkpoint, the spindle assembly checkpoint or SAC [30]. As long as chromosomes are not correctly attached with their kinetochores to the bipolar spindle, the SAC is active and prevents cell cycle progression. The ensuing cell cycle delay permits the establishment of correct attachments before chromosome segregation takes place [31]. Because securin degradation depends on the satisfaction of the SAC, it is thereby ensured that separase does not become activated before all chromosomes are correctly attached.

Importantly, securin is in excess over separase. If securin binds with high affinity to separase, the excess of securin guarantees that even when the APC/C becomes active, separase is kept inhibited as long as there is enough free securin around. Alternatively, it was proposed that PP2A-B56 bound to separase keeps associated securin in an unphosphorylated state. In this model, it was proposed that the APC/C would have a higher affinity towards phosphorylated -thus free- securin, thereby ensuring degradation of free securin before separase-bound securin [16,32,33]. 

Given the crucial role of securin for separase inhibition, it came as a surprise that mice invalidated for securin are viable, and cultured cells depleted for securin divide without major problems, showing that securin is not essential to ensure genome stability in most tissues [34,35,36,37]. However, this can be explained by the fact that securin is not the only inhibitory mechanism in place to control separase.

#### 2.1.2. Cyclin B1

The second inhibitory mechanism keeping separase in check involves cyclin B1/Cdk1 together with the small accessory protein Cks1. Cdk1, in association with M-phase cyclins, constitutes the master cell cycle kinase driving progression through mitosis and meiosis. Cyclin B1/Cdk1 phosphorylates a serine residue in separase, which mediates binding of cyclin B1/Cdk1 to separase and its mutual inhibition [38,39,40,41]. Through structural studies, it was discovered that separase-bound cyclin B1/Cdk1 together with Cks1 fixes three autoinhibitory loops in separase, thus blocking the binding of Scc1 or securin to recognition motifs in separase [27]. Accordingly, separase binding to securin or cyclinB1/Cdk1 is mutually exclusive [39]. The binding of cyclin B1 to the residue in separase that cyclin B1/Cdk1 itself has phosphorylated requires a very recently identified cyclin B1 phosphate-binding pocket [27].

Like securin, cyclin B1 degradation depends on SAC satisfaction and APC/C-dependent degradation [30,31]. Activation of the APC/C thus removes both separase inhibitors, securin and cyclin B1. However, whereas securin is completely degraded at anaphase onset, cyclin B1 degradation takes place in two steps, resulting in the complete degradation and loss of associated kinase activity only after anaphase, to exit mitosis [42,43,44]. Most separase is inhibited by securin, and only around 6% of total separase is found to interact with cyclin B1 in arrested metaphase cells [25]. In the presence of wild-type levels of securin, cyclin B1’s role becomes important once securin has been degraded to prevent precocious re-activation of separase at the end of mitosis, which would interfere with the reloading of cohesin complexes. Phosphorylation of separase by cyclin B1 allows the peptidyl-prolyl-isomerase Pin1 to convert separase from a trans to a cis conformation. This cis-confirmation was proposed to be inhibited at the end of mitosis only by cyclin B1 and only when cyclin B1 is unphosphorylated and in association with Cdk1 [25,45]. More recently, however, Pin1 was found to be dispensable for the cyclin B1/Cdk1-Separase interaction in vitro [27], putting into question the role of cis-or trans-isomerization of the specific proline residue targetted by Pin1. In summary, cyclin B1’s main role as a separase inhibitor seems to be preventing too early re-activation of separase at anaphase-telophase. Cyclin B1/Cdk1 can take over -at least partially- the role of securin when it is absent, but this may not be enough to completely inhibit separase (see below). How cyclin B1/Cdk1 can take over in metaphase when securin is absent, when under normal circumstances its inhibitory role on separase is restrained to late anaphase, still needs to be clarified.

#### 2.1.3. Sgo2/Mad2

A third separase inhibitor, namely Sgo2/Mad2, has been identified recently in mitotic tissue culture cells [25]. The requirement for a third inhibitor was suggested by the results below, together difficult to reconcile with only securin and cyclinB1/Cdk1 keeping separase in check: (1) cells without securin are able to activate separase on time and segregate chromosomes; (2) cells without the APC/C activator Cdc20 arrest in metaphase without chromosome segregation, because they cannot degrade cyclin B1 to decrease Cdk1 activity below the threshold required for anaphase onset; (3) however, cells that are devoid of both, Cdc20 and securin, arrest in metaphase but now with segregated chromosomes, even though cyclin B1/Cdk1 as separase inhibitor is present [25]. Prior to activating the APC/C, Cdc20 is part of the MCC (mitotic checkpoint complex) which maintains cells arrested when the SAC is active [31]. The phenotype upon loss of both Cdc20 and securin indicates that, surprisingly, the inhibition of separase by cyclin B1 is not functional under these conditions, and inhibition of separase requires the APC/C activator Cdc20. Based on these data, the SAC was proposed to be involved in separase inhibition beyond APC/C inhibition. A recent publication showed that separase could be kept inactive by Mad2 (essential for the SAC and component of the MCC) together with Sgo2 [25]. For anaphase onset, the SAC is inactivated through the disassembly of the MCC by the action of the AAA^+^ ATPase TRIP13 and the co-factor p31Comet [46]. The same mechanism seems to apply for breaking the inhibitory interaction between separase and Sgo2/Mad2 [25].

#### 2.1.4. Limitations

All three inhibitory mechanisms impinging on separase in mitosis are not essential on their own. In somatic tissue culture cells that are arrested in metaphase by activating the SAC with taxol, most separase is inhibited by securin, followed by Sgo2/Mad2 and finally cyclin B1/Cdk1 [25]. Keeping cells arrested by activating the SAC may enhance the contribution of a SAC-dependent mechanism controlling separase. Prolonged SAC activation and release from prolonged SAC arrest also leads to the presence of high cyclin B1/Cdk1 activity, leading to more phosphorylated Cyclin B1 with a much lower affinity for separase than its unphosphorylated form. Hence, more cyclin B1 may be associated with separase in unchallenged metaphase cells. It remains to be determined if the ratio of the different inhibitors stays the same in unchallenged cycling cells that progress through mitosis.

It is likely that the relative importance of each inhibitory pathway also varies in vivo depending on the cell type. It was shown in mice that complete loss of securin does not affect survival and gametogenesis in any obvious manner [35,37]. Heterozygote mice and embryonic stem cells harboring one allele of separase that cannot be phosphorylated by cyclin B1 are alive without any overt phenotype. However, mice are sterile because embryonic germ cells are not viable. Early embryonic divisions are equally affected by the presence of this one allele of separase that cannot be phosphorylated and thus inhibited by cyclin B1/Cdk1, leading to embryonic lethality. Interestingly, securin levels are much lower during very early embryogenesis, indicating that proper separase inhibition may depend on the equilibrium of the different inhibitory mechanisms in each cell type [47,48,49].

#### 2.1.5. Separase Inactivation at the Exit from Mitosis

Exit from mitosis is characterized by a complete loss of Cdk1 activity. The next cell cycle starts with the appearance of G1 cyclins and a slow increase in Cdk activities with the synthesis of cell-stage specific cyclins, allowing cells to progress from G1 into S-phase and G2. During this time, separase from the previous cycle might be kept inactive due to the conformational change induced by Pin1, whereas newly synthesized separase should be co-translated with securin and hence, inhibited. Separase is furthermore physically separated from chromosomes due to an NES (nuclear export signal), localizing separase outside the nucleus to the cytoplasm during interphase [50]. However, it is still unclear whether there are other mechanisms keeping separase under control outside mitosis.

### 2.2. Separase Control in Meiosis

**Figure 2:**
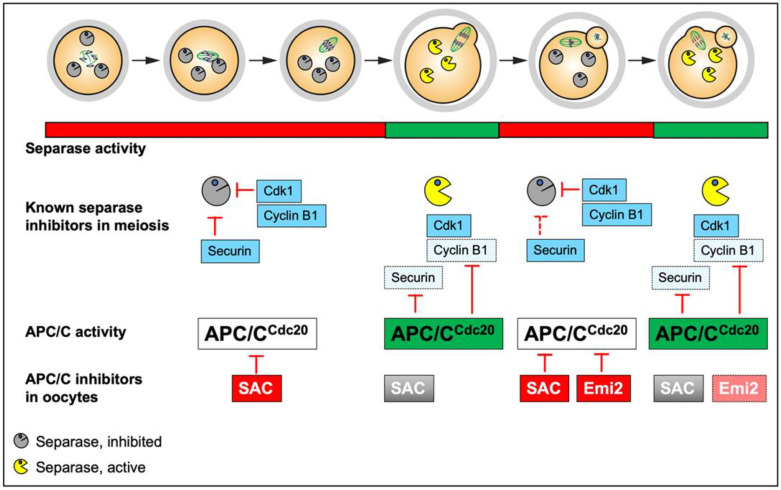
Inhibitory mechanisms controlling separase that have been described in oocyte meiosis. Separase undergoes two activation cycles throughout the two meiotic divisions, indicated in red (inactive) and green (active). Two mechanisms have been shown for up to now to keep separase under control in mouse oocytes: cyclin B1-Cdk1 dependent phosphorylation of separase and binding to securin. Both cyclin B1 and securin are targets of APC/C^Cdc20^ -dependent ubiquitination followed by their degradation, separase activation thus depends on APC/C^Cdc20^ activation (indicated by the green color). In oocytes, APC/C^Cdc20^ activity is under the control of the SAC, which is inactivated once error-free attachments of chromosomes to the bipolar spindle have been achieved, and Emi2, which inhibits the APC/C^Cdc20^ in meiosis II until fertilization occurs, inducing degradation of Emi2. Lighter shades of the same entity indicate inactivation or degradation.

In meiosis, separase has to be activated twice: once in meiosis I, to remove cohesin from chromosome arms and immediately afterward in meiosis II, to cleave pericentromeric cohesin. The fact that there is no intervening S-phase, no reformation of the nuclear envelope to physically separate separase from chromosomes, and no reset of the cell cycle poses additional challenges to separase regulation. In both meiosis I and meiosis II, separase activation requires APC/C activation [51,52]. In meiosis II, however, an additional challenge for separase activation in oocytes comes from the need to remain arrested in CSF-arrest for hours to days (mouse and human oocytes, respectively). CSF-arrest is mediated through the inhibition of the APC/C by the CSF-factor Emi2, an inhibitor of the APC/C and degraded upon fertilization [52,53]. Separase is thus activated at fertilization because the APC/C is no longer inhibited (Figure 2).

Live imaging of a separase biosensor harboring the mitotic cohesin subunit Scc1 as a separase substrate indicated that separase is activated in metaphase of meiosis I before being inactivated again as oocytes progress into meiosis II [54]. Securin and cyclin B1 are degraded in an APC/C-dependent manner, with cyclin B1 degradation that continues throughout anaphase I [55,56,57,58,59]. APC/C activation depends on the SAC, and in the absence of SAC control, securin and cyclin B1 are degraded precociously, leading to separase activation before all chromosomes have been correctly attached and hence, to aneuploidies in the resulting gamete [60,61,62,63]. However, several open questions remain concerning separase control in meiosis I, its tight inactivation as cells exit meiosis I, and separase re-activation in meiosis II only upon fertilization.

#### 2.2.1. Securin

Female mice devoid of securin are fertile, indicating that securin is not essential for separase control during oocyte meiosis [35,37]. Nevertheless, it has been proposed that it is predominantly securin that keeps separase in check in meiosis II [64]. Knock-down of securin led to sister chromatid segregation in metaphase II in one study; however, this is not compatible with the fact that complete loss of securin in knock-out mice does not affect female fertility [65]. Complete APC/C-dependent degradation of securin is a condition for separase activation and chromosome segregation in oocytes (Figure 2) [55,56]. Like in mitosis, securin was found to be in excess over separase in meiosis I in mouse oocytes, with free securin not associated with separase being degraded prior to separase-bound securin. Separase binding masks two residues in securin which are required for free securin to be degraded. This mechanism seems to be necessary to ensure that securin inhibiting separase is degraded last but that there is also enough securin around to inhibit all separase present. Indicating that this mechanism indeed has a physiological role, the absence of free securin degradation prior to metaphase I perturbs entry into anaphase I [66].

#### 2.2.2. Cyclin B1

Similar to securin, also cyclin B1 is in excess relative to its partner Cdk1 in oocytes. Again, free cyclin B1 is degraded prior to Cdk1-bound cyclin B1 in an APC/C-dependent manner in metaphase I. Cdk1 binding to cyclin B1 masks a degron motif required for prometaphase but not metaphase destruction of cyclin B1 in oocyte meiosis I [59]. Like in mitosis, separase is kept in check by cyclin B1/Cdk1 in meiosis I and probably also meiosis II (Figure 2), at least when securin is absent [35,37]. In meiosis, I, expression of a separase mutant that cannot be phosphorylated by cyclinB1/Cdk1 does not lead to precocious sister separation in oocytes, as expected if cyclin B1-dependent phosphorylation of separase were the only mechanism keeping separase in check [65,67]. The combination of this non-phosphorylatable mutant with securin knockdown lead to sister separation before metaphase-to-anaphase transition in meiosis I in one study, however under conditions where securin knock-down on its own -unlike complete loss of securin- resulted in sister separation in meiosis II [65]. It still remains to be addressed whether securin becomes essential in meiosis I in this context, using a securin knock-out approach.

#### 2.2.3. Pin1 and Sgo2/Mad2

The potential roles of Pin1 and/or Sgo2/Mad2 as separase inhibitors in meiosis have not been addressed yet. It is worthwhile mentioning that the interaction of Sgo2 with Mad2 was found to be involved in SAC silencing in mouse oocyte meiosis I [12]. Hence, Sgo2/Mad2 promotes activation of separase through APC/C-dependent degradation of securin and cyclin B1, making it seem counterintuitive that at the same time, Sgo2/Mad2 would inhibit separase. Future work will clarify the role of Sgo2/Mad2 in oocyte meiosis. Whether Pin1 has a physiological role in the transition from meiosis I to meiosis II and/or at exit from meiosis II is another open issue that needs to be addressed in the future.

## 3. The Meiotic Cohesin Subunit Rec8

Rec8 on chromosome arms is cleaved in meiosis I, and only a tiny fraction of Rec8 at the pericentromere in meiosis II [1,11]. How separase is prevented from cleaving this small fraction of Rec8 in the centromere region is still far from clear in higher eukaryotes. Sgo2 at centromeres is required for protecting Rec8 from separase, and it does so by localizing PP2A-B56 to the centromere region [12,13,14,68]. Mice harboring a complete knock-out of Sgo2 are viable without any discernable phenotype, except that they are sterile because they cannot generate gametes with the correct haploid chromosome count [13]. This is due to the fact that without Sgo2, all Rec8 is cleaved in meiosis I, leading to precocious sister chromatid separation in meiosis I. Upon progression into meiosis II, separated sister chromatids cannot establish correct tension-bearing attachments to the opposite poles of the bipolar spindle and thus segregate at random.

### 3.1. Rec8 Phosphorylation for Cleavage in Lower Eukaryotes

The fact that PP2A-B56 centromeric localization through Sgo2 is required for the protection of pericentromeric cohesin in mammalian meiosis I indicated that phosphorylation plays an important role in promoting Rec8 cleavage [10]. Indeed, in both budding and fission yeast, phosphorylation of Rec8 by Casein kinase 1 (CK1, both budding and fission yeast) and Cdc7/Dbf4 (budding yeast) was previously shown to be required for its cleavage [69,70,71]. Substituting the residues identified with non-phosphorylatable Alanine residues abrogates cleavage in vivo and, as a consequence, prevents chromosome segregation. *C. elegans* Rec8 equally needs to be phosphorylated for cleavage in meiosis I, but by a different kinase, namely Aurora B [72,73]. However, whether mammalian Rec8 equally requires phosphorylation for cleavage in vivo remained enigmatic until recently.

### 3.2. Aurora B/C Kinases Phosphorylate Rec8 in Mammalian Oocytes for Separase-Dependent Cleavage

Mammalian germ cells express three different Aurora kinases: Aurora A and the very closely to each other related Aurora B and C [74]. Whereas loss of Aurora A leads to metaphase I arrest with SAC activation, loss of both Aurora B and C results in cytokinesis failures in oocytes. Even though oocytes cannot properly extrude a polar body in the absence of Aurora B/C, they undergo anaphase I. However, they are not able to segregate chromosomes correctly because Rec8 is not properly removed from chromosome arms. Using a separase biosensor adapted to study cleavage of various phosphomutants of mouse Rec8, we identified Serine 482 as a phosphorylation site required for Rec8 sensor cleavage in oocytes. Accordingly, this phosphosite corresponds to an Aurora B/C consensus site and is phosphorylated by Aurora B in vitro. Importantly, endogenous Rec8 is indeed phosphorylated on Serine 482 in an Aurora B/C-dependent manner [11]. Hence, mammalian Rec8 needs to be phosphorylated for cleavage by separase. Aurora B/C may additionally phosphorylate other sites than the one identified up to now, and it remains to be determined whether other kinases such as CK1δ or Cdc7/Dbf4 also promote mammalian Rec8 cleavage.

## 4. Cohesin Protection and Deprotection

If phosphorylation of Rec8 brings about its cleavage on arms in meiosis I, it is attractive to speculate that counteracting this phosphorylation prevents and thus protects Rec8 from being cleaved at the pericentromere. Allowing phosphorylation of Rec8 at this location in meiosis II would then lead to the deprotection of pericentromeric cohesin and its cleavage at anaphase II onset. This model turned out to be true in yeast [75,76]; however, whether the same mechanism applies to mammalian oocytes is extremely difficult to address and thus still unknown. Cohesive cohesin complexes are loaded after premeiotic S-phase, before birth of the female, whereas cohesin cleavage by separase takes place at reproductive maturity in the adult [77]. Hence, it is impossible to study requirements for cohesin removal by transient approaches (such as expressing Rec8 mutants upon resumption of meiosis) because components of the cohesin complex expressed after S-phase are not integrated into existing cohesin complexes to be cohesive. The investment in time and research funds to generate mouse models required to properly study cohesive Rec8 cleavage by a mutant approach has been prohibitory to determine which post-translational modifications of centromeric Rec8 bring about its protection in meiosis I.

Using phospho-specific antibodies recognizing Rec8 phosphorylated on S482, my group hoped to be able to detect the fraction of Rec8 not being phosphorylated through the absence of the signal when compared to total Rec8 staining, but this was not the case [11]. This is due to the fact that only a very small fraction of Rec8 in the centromere region is not cleaved in meiosis I; the bulk of Rec8 localized there is unprotected and hence, still phosphorylated and cleaved. The fraction of protected Rec8 at the pericentromere may be buried beneath unprotected Rec8 and hence, impossible to detect by classical antibody staining.

If we assume that removing phosphosites on Rec8 brings about protection in meiosis I, the next question arising is the issue of how phosphorylation is then permitted in meiosis II to allow segregation of sister chromatids. Alternatively, Rec8 cleavage at the centromere may not depend on phosphorylation in meiosis II in higher eukaryotes, because some other, still unknown post-translational modification is required, or some inhibitory protein interaction interfering with cleavage has been removed. Below, I summarize our current knowledge of cohesin cleavage in meiosis II.

### 4.1. No Tension-Dependent Removal of Proteins Protecting Centromeric Cohesin

Chromosomes consisting of two sister chromatids each are attached to the opposite poles of the bipolar spindle in meiosis I, and sister chromatids in meiosis II. Sister kinetochores are thus co-oriented in meiosis I and in a back-to-back configuration in meiosis II. The back-to-back orientation leads to the stretching-apart of sister kinetochores attached to microtubules which tear them to opposite poles, whereas in meiosis I, sister kinetochores seem to function as one unit, and it is the chromosomes themselves that are stretched apart. It was attractive to propose that the bipolar stretch experienced by sister kinetochores in meiosis II would move proteins preventing Rec8 phosphorylation (Sgo2 recruiting PP2A-B56) away from the centromeric region where Rec8 is holding sister chromatids together [68,78]. However, this turned out not to be the case: PP2A-B56 is found to still co-localize with centromeric cohesin in meiosis II, and pericentromeric cohesin is still cleaved in oocytes with sister chromatids that are not under bipolar, but monopolar tension [15,79,80]. Hence, bipolar tension is not a condition for centromeric cohesin cleavage in meiosis II.

### 4.2. Is There Cohesin Protection by Sgo2 in Meiosis II?

In mammalian meiosis I, Sgo2, together with PP2A-B56, is required for centromeric cohesin protection. However, Sgo2 also counteracts Aurora B/C kinase activity in a pathway involved in correcting wrong microtubule attachments, by silencing the SAC through its interaction with Mad2; and finally, moderating the tension applied by the bipolar spindle [12]. Both Aurora B/C kinase downregulation and silencing of the SAC require the interaction of Sgo2 with PP2A-B56, making it difficult to distinguish which pool of Sgo2 is required for cohesin protection and which pool fulfills other roles. Sgo2 is recruited through two kinases, Bub1 and Mps1, to the centromere region [81]. Through loss of function mouse models combined with specific kinase inhibitors, it was shown that Sgo2 recruited by Mps1 brings about cohesin protection in meiosis I. Accordingly, inhibition of Mps1 but not Bub1 kinase activity leads to precocious sister chromatid segregation in meiosis I. Hence, it is only this Mps1-recruited pool of Sgo2 which ensures that separase cannot access the centromeric fraction of Rec8 maintained until meiosis II [81]. Intriguingly, the Mps1-dependent fraction of Sgo2 is also recruited in meiosis II, where Rec8 should not be protected anymore. It remains to be addressed whether protection remains in place until anaphase II onset and whether Sgo2 protects Rec8 in meiosis II as well. It is still unknown when exactly at the transition from meiosis I to meiosis II deprotection in oocytes actually takes place, and indeed, protection may well be in place until anaphase II. Alternatively, Mps1-recruited Sgo2 may occupy other roles in meiosis II, unrelated to cohesin protection. Whatever Sgo2 is doing there, it is not essential for cohesin protection until separase becomes activated for anaphase II onset. Indeed, oocytes devoid of Mps1 kinase activity are able to maintain a cell cycle arrest awaiting fertilization without precocious sister chromatid separation in meiosis II [82].

In *S. cerevisiae*, Mps1 and Sgo1 (which protects centromeric cohesin in meiosis I and is colocalizing with Rec8 in meiosis II) are degraded prior to anaphase II onset in an APC/C-dependent manner [75]. In mouse oocytes, Mps1 and Sgo2 can still be found associated with kinetochores on separating anaphase II chromatids, excluding that degradation alone of both proteins is necessary for deprotection [79]. Thus, it is still open whether Sgo2 is required to protect centromeric cohesin in meiosis II until anaphase II onset, and if yes, how this protective mechanism is inactivated to allow separase cleavage of centromeric cohesin for sister chromatid segregation. It remains to be addressed whether a protective mechanism mediated by Sgo2 serves as some kind of security system in case proper inhibition of separase fails during the prolonged oocyte arrest to await fertilization, which is not the case under normal conditions.

### 4.3. Accessibility of Centromeric Rec8

**Figure 3:**
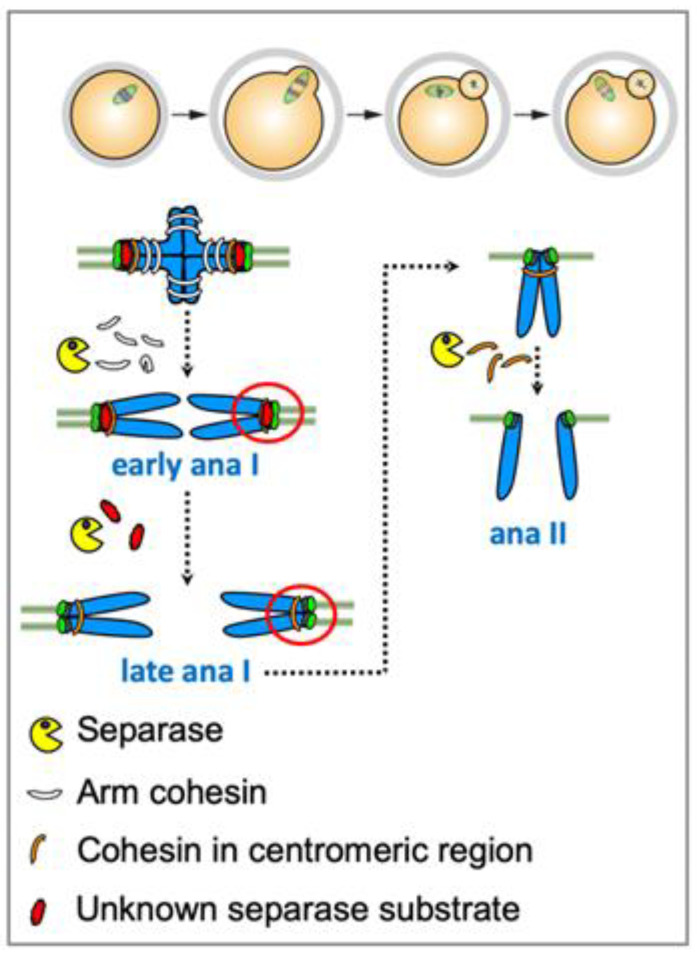
Kinetochore individualization in meiosis I is required for centromeric cohesin removal in meiosis II. Arm cohesin removal at the metaphase-to-anaphase transition in meiosis I is followed by separase-dependent removal of the physical connections holding sister kinetochores together as one unit. The protein(s) fusing sister kinetochores together that has/have to be cleaved in anaphase I is still unknown. Separase-dependent cleavage giving rise to the individualization of sister kinetochores (indicated with a red circle) is a prerequisite for cleavage of pericentromeric cohesin in meiosis II. Artificially introduced paired chromosomes in meiosis II segregate into chromosomes and not sister chromatids, but only when there was no prior sister kinetochore individualization.

Kinetochores appear as fused together in meiosis I and are thought to behave as one unit instead of two. Maybe Rec8 protection in oocyte meiosis I is due to the fact that Rec8 kinases cannot access the pool of Rec8 at the pericentromere that needs to maintain sister chromatids together in meiosis II? A recently described event called “kinetochore individualization” [79] indicates that this hypothesis deserves consideration (Figure 3). It was shown that the physical separation of sister kinetochores at the transition from meiosis I to meiosis II is not due to the fact that attachments change from monopolar to bipolar but to separase-dependent cleavage of a yet-to-be-identified protein in anaphase I. Without this physical separation of sister kinetochores in meiosis I, sister chromatids cannot segregate in meiosis II. Attractive candidates for separase substrates that are responsible for fusing sister kinetochores together are a protein called Meikin [83,84] and/or an additional, separate fraction of Rec8. Meikin was identified through its interaction with the centromere protein CenPC and shown to promote mono-orientation. In early prometaphase I, sister kinetochores in Meikin knock-out oocytes are further apart from each other than in control oocytes. However, oocytes devoid of Meikin show fused sister kinetochores in metaphase I, and it is only in anaphase I that kinetochores seem to individualize earlier or more efficiently than in control oocytes. Kinetochore fusion may thus be promoted by additional factors, with Meikin contributing but not being the only factor involved [83]. Future work will show how protection by Sgo2-PP2A-B56, kinetochore individualization, and a protein called I2PP2A/Set, which was shown to promote cohesin cleavage in meiosis II [15], work together for timely centromeric cohesin removal and sister chromatid segregation in meiosis II.

## 5. Perspectives

Mammalian oocytes are generated before birth in females and remain arrested in prophase I for an extended time until sexual maturity. In humans, this delay can last for more than 40 years. During all this time, chromosomes are held together by the cohesin complex, which is not renewed - the proteins are thus as old as the female. Not surprisingly, given the overall duration of female meiosis, cohesion gets weaker with advancing maternal age, destabilizing chiasmata and centromeric cohesion, essential for correct tension-bearing attachments to the spindle in meiosis I and II. The rate of precocious separation of chromosomes and sister chromatids is hence increased with age. In humans, this is in part responsible for the elevated error rates in female meiosis with maternal age. Missegregations often concern the smaller chromosomes, which have only one chiasma and are, therefore, the most affected when this only chiasma is weakened due to cohesin loss. In humans, the most frequent viable trisomy -Trisomy 21- is in around 90% of cases due to missegregations in oocyte meiosis I, and chromosome 21 is also one of the smallest chromosomes [2,51,77]. Better knowledge of separase control and Rec8 cleavage will not only help us to understand these age-related aneuploidies, but insights gained into this process may potentially allow the identification of risk factors and be the basis for individual counseling in the clinic.

## Data Availability

Not applicable.

## References

[1] Petronczki M., Siomos M.F., Nasmyth K. (2003). Un menage a quatre: The molecular biology of chromosome segregation in meiosis. Cell.

[2] MacLennan M., Crichton J.H., Playfoot C.J., Adams I.R. (2015). Oocyte development, meiosis and aneuploidy. Semin. Cell Dev. Biol..

[3] Mihajlovic A.I., FitzHarris G. (2018). Segregating Chromosomes in the Mammalian Oocyte. Curr. Biol..

[4] Ur S.N., Corbett K.D. (2021). Architecture and Dynamics of Meiotic Chromosomes. Annu. Rev. Genet..

[5] Grey C., de Massy B. (2021). Chromosome Organization in Early Meiotic Prophase. Front. Cell Dev. Biol..

[6] Pyatnitskaya A., Borde V., De Muyt A. (2019). Crossing and zipping: Molecular duties of the ZMM proteins in meiosis. Chromosoma.

[7] Moore D.P., Orr-Weaver T.L. (1998). Chromosome segregation during meiosis: Building an unambivalent bivalent. Curr. Top. Dev. Biol..

[8] Lister L.M., Kouznetsova A., Hyslop L.A., Kalleas D., Pace S.L., Barel J.C., Nathan A., Floros V., Adelfalk C., Watanabe Y. (2010). Age-related meiotic segregation errors in Mammalian oocytes are preceded by depletion of cohesin and Sgo2. Curr. Biol..

[9] Hodges C.A., Revenkova E., Jessberger R., Hassold T.J., Hunt P.A. (2005). SMC1beta-deficient female mice provide evidence that cohesins are a missing link in age-related nondisjunction. Nat. Genet..

[10] Marston A.L., Wassmann K. (2017). Multiple Duties for Spindle Assembly Checkpoint Kinases in Meiosis. Front. Cell Dev. Biol..

[11] Nikalayevich E., El Jailani S., Dupre A., Cladiere D., Gryaznova Y., Fosse C., Buffin E., Touati S.A., Wassmann K. (2022). Aurora B/C-dependent phosphorylation promotes Rec8 cleavage in mammalian oocytes. Curr. Biol..

[12] Rattani A., Wolna M., Ploquin M., Helmhart W., Morrone S., Mayer B., Godwin J., Xu W., Stemmann O., Pendas A. (2013). Sgol2 provides a regulatory platform that coordinates essential cell cycle processes during meiosis I in oocytes. eLife.

[13] Llano E., Gomez R., Gutierrez-Caballero C., Herran Y., Sanchez-Martin M., Vazquez-Quinones L., Hernandez T., de Alava E., Cuadrado A., Barbero J.L. (2008). Shugoshin-2 is essential for the completion of meiosis but not for mitotic cell division in mice. Genes Dev..

[14] Mailhes J.B., Hilliard C., Fuseler J.W., London S.N. (2003). Okadaic acid, an inhibitor of protein phosphatase 1 and 2A, induces premature separation of sister chromatids during meiosis I and aneuploidy in mouse oocytes in vitro. Chromosome Res. Int. J. Mol. Supramol. Evol. Asp. Chromosome Biol..

[15] Chambon J.P., Touati A.S., Berneau S., Hebras C., Groeme R., Cladière D., Dumollard R., McDougall A., Wassmann K. (2013). The PP2A inhibitor I2PP2A is essential for sister chromatid segregation in meiosis II. Curr. Biol..

[16] Kamenz J., Hauf S. (2017). Time To Split Up: Dynamics of Chromosome Separation. Trends Cell Biol..

[17] Rosen L.E., Klebba J.E., Asfaha J.B., Ghent C.M., Campbell M.G., Cheng Y., Morgan D.O. (2019). Cohesin cleavage by separase is enhanced by a substrate motif distinct from the cleavage site. Nat. Commun..

[18] Waizenegger I.C., Hauf S., Meinke A., Peters J.M. (2000). Two distinct pathways remove mammalian cohesin from chromosome arms in prophase and from centromeres in anaphase. Cell.

[19] Nishiyama T., Sykora M.M., Veld P.J.H.I., Mechtler K., Peters J.M. (2013). Aurora B and Cdk1 mediate Wapl activation and release of acetylated cohesin from chromosomes by phosphorylating Sororin. Proc. Natl. Acad. Sci. USA.

[20] Tedeschi A., Wutz G., Huet S., Jaritz M., Wuensche A., Schirghuber E., Davidson I.F., Tang W., Cisneros D.A., Bhaskara V. (2013). Wapl is an essential regulator of chromatin structure and chromosome segregation. Nature.

[21] Nishiyama T., Ladurner R., Schmitz J., Kreidl E., Schleiffer A., Bhaskara V., Bando M., Shirahige K., Hyman A.A., Mechtler K. (2010). Sororin mediates sister chromatid cohesion by antagonizing Wapl. Cell.

[22] Gandhi R., Gillespie P.J., Hirano T. (2006). Human Wapl is a cohesin-binding protein that promotes sister-chromatid resolution in mitotic prophase. Curr. Biol..

[23] Shintomi K., Hirano T. (2009). Releasing cohesin from chromosome arms in early mitosis: Opposing actions of Wapl-Pds5 and Sgo1. Genes Dev..

[24] Kudo N.R., Wassmann K., Anger M., Schuh M., Wirth K.G., Xu H., Helmhart W., Kudo H., McKay M., Maro B. (2006). Resolution of chiasmata in oocytes requires separase-mediated proteolysis. Cell.

[25] Hellmuth S., Gomez H.L., Pendas A.M., Stemmann O. (2020). Securin-independent regulation of separase by checkpoint-induced shugoshin-MAD2. Nature.

[26] Waizenegger I., Gimenez-Abian J.F., Wernic D., Peters J.M. (2002). Regulation of human separase by securin binding and autocleavage. Curr. Biol..

[27] Yu J., Raia P., Ghent C.M., Raisch T., Sadian Y., Cavadini S., Sabale P.M., Barford D., Raunser S., Morgan D.O. (2021). Structural basis of human separase regulation by securin and CDK1-cyclin B1. Nature.

[28] Lin Z., Luo X., Yu H. (2016). Structural basis of cohesin cleavage by separase. Nature.

[29] Hellmuth S., Pohlmann C., Brown A., Bottger F., Sprinzl M., Stemmann O. (2015). Positive and negative regulation of vertebrate separase by Cdk1-cyclin B1 may explain why securin is dispensable. J. Biol. Chem..

[30] Alfieri C., Zhang S., Barford D. (2017). Visualizing the complex functions and mechanisms of the anaphase promoting complex/cyclosome (APC/C). Open Biol..

[31] Etemad B., Kops G.J. (2016). Attachment issues: Kinetochore transformations and spindle checkpoint silencing. Curr. Opin Cell Biol..

[32] Hellmuth S., Bottger F., Pan C., Mann M., Stemmann O. (2014). PP2A delays APC/C-dependent degradation of separase-associated but not free securin. EMBO J..

[33] Shindo N., Kumada K., Hirota T. (2012). Separase sensor reveals dual roles for separase coordinating cohesin cleavage and cdk1 inhibition. Dev. Cell.

[34] Jallepalli P.V., Waizenegger I.C., Bunz F., Langer S., Speicher M.R., Peters J.M., Kinzler K.W., Vogelstein B., Lengauer C. (2001). Securin is required for chromosomal stability in human cells. Cell.

[35] Mei J., Huang X., Zhang P. (2001). Securin is not required for cellular viability, but is required for normal growth of mouse embryonic fibroblasts. Curr. Biol..

[36] Pfleghaar K., Heubes S., Cox J., Stemmann O., Speicher M.R. (2005). Securin is not required for chromosomal stability in human cells. PLoS Biol..

[37] Wang Z., Yu R., Melmed S. (2001). Mice lacking pituitary tumor transforming gene show testicular and splenic hypoplasia, thymic hyperplasia, thrombocytopenia, aberrant cell cycle progression, and premature centromere division. Mol. Endocrinol..

[38] Holland A.J., Taylor S.S. (2006). Cyclin-B1-mediated inhibition of excess separase is required for timely chromosome disjunction. J. Cell Sci..

[39] Gorr I.H., Boos D., Stemmann O. (2005). Mutual inhibition of separase and Cdk1 by two-step complex formation. Mol. Cell.

[40] Stemmann O., Zou H., Gerber S.A., Gygi S.P., Kirschner M.W. (2001). Dual inhibition of sister chromatid separation at metaphase. Cell.

[41] Huang X., Hatcher R., York J.P., Zhang P. (2005). Securin and separase phosphorylation act redundantly to maintain sister chromatid cohesion in mammalian cells. Mol. Biol. Cell.

[42] Afonso O., Castellani C.M., Cheeseman L.P., Ferreira J.G., Orr B., Ferreira L.T., Chambers J.J., Morais-de-Sa E., Maresca T.J., Maiato H. (2019). Spatiotemporal control of mitotic exit during anaphase by an aurora B-Cdk1 crosstalk. eLife.

[43] Collin P., Nashchekina O., Walker R., Pines J. (2013). The spindle assembly checkpoint works like a rheostat rather than a toggle switch. Nat. Cell Biol..

[44] Wolf F., Wandke C., Isenberg N., Geley S. (2006). Dose-dependent effects of stable cyclin B1 on progression through mitosis in human cells. EMBO J..

[45] Hellmuth S., Rata S., Brown A., Heidmann S., Novak B., Stemmann O. (2015). Human chromosome segregation involves multi-layered regulation of separase by the peptidyl-prolyl-isomerase Pin1. Mol. Cell.

[46] Vader G. (2015). Pch2(TRIP13): Controlling cell division through regulation of HORMA domains. Chromosoma.

[47] Huang X., Andreu-Vieyra C.V., Wang M., Cooney A.J., Matzuk M.M., Zhang P. (2009). Preimplantation mouse embryos depend on inhibitory phosphorylation of separase to prevent chromosome missegregation. Mol. Cell. Biol..

[48] Huang X., Andreu-Vieyra C.V., York J.P., Hatcher R., Lu T., Matzuk M.M., Zhang P. (2008). Inhibitory phosphorylation of separase is essential for genome stability and viability of murine embryonic germ cells. PLoS Biol..

[49] Xu J., Wang M., Gao X., Hu B., Du Y., Zhou J., Tian X., Huang X. (2011). Separase phosphosite mutation leads to genome instability and primordial germ cell depletion during oogenesis. PLoS ONE.

[50] Sun Y., Yu H., Zou H. (2006). Nuclear exclusion of separase prevents cohesin cleavage in interphase cells. Cell Cycle.

[51] Holt J.E., Lane S.I., Jones K.T. (2013). The control of meiotic maturation in mammalian oocytes. Curr. Top. Dev. Biol..

[52] Wu J.Q., Kornbluth S. (2008). Across the meiotic divide—CSF activity in the post-Emi2/XErp1 era. J. Cell Sci..

[53] Schmidt A., Rauh N.R., Nigg E.A., Mayer T.U. (2006). Cytostatic factor: An activity that puts the cell cycle on hold. J. Cell Sci..

[54] Nikalayevich E., Bouftas N., Wassmann K. (2018). Detection of Separase Activity Using a Cleavage Sensor in Live Mouse Oocytes. Methods Mol. Biol..

[55] Herbert M., Levasseur M., Homer H., Yallop K., Murdoch A., McDougall A. (2003). Homologue disjunction in mouse oocytes requires proteolysis of securin and cyclin B1. Nat. Cell Biol..

[56] Terret M.E., Wassmann K., Waizenegger I., Maro B., Peters J.M., Verlhac M.H. (2003). The Meiosis I-to-Meiosis II Transition in Mouse Oocytes Requires Separase Activity. Curr. Biol..

[57] Ledan E., Polanski Z., Terret M.E., Maro B. (2001). Meiotic maturation of the mouse oocyte requires an equilibrium between cyclin B synthesis and degradation. Dev. Biol..

[58] Karasu M.E., Bouftas N., Keeney S., Wassmann K. (2019). Cyclin B3 promotes anaphase I onset in oocyte meiosis. J. Cell Biol..

[59] Levasseur M.D., Thomas C., Davies O.R., Higgins J.M.G., Madgwick S. (2019). Aneuploidy in Oocytes Is Prevented by Sustained CDK1 Activity through Degron Masking in Cyclin B1. Dev. Cell.

[60] Wassmann K., Niault T., Maro B. (2003). Metaphase I Arrest upon Activation of the Mad2-Dependent Spindle Checkpoint in Mouse Oocytes. Curr. Biol..

[61] Homer H.A., McDougall A., Levasseur M., Murdoch A.P., Herbert M. (2005). Mad2 is required for inhibiting securin and cyclin B degradation following spindle depolymerisation in meiosis I mouse oocytes. Reproduction.

[62] Homer H.A., McDougall A., Levasseur M., Yallop K., Murdoch A.P., Herbert M. (2005). Mad2 prevents aneuploidy and premature proteolysis of cyclin B and securin during meiosis I in mouse oocytes. Genes Dev..

[63] Hached K., Xie S.Z., Buffin E., Cladiere D., Rachez C., Sacras M., Sorger P.K., Wassmann K. (2011). Mps1 at kinetochores is essential for female mouse meiosis I. Development.

[64] Nabti I., Reis A., Levasseur M., Stemmann O., Jones K.T. (2008). Securin and not CDK1/cyclin B1 regulates sister chromatid disjunction during meiosis II in mouse eggs. Dev. Biol..

[65] Chiang T., Schultz R.M., Lampson M.A. (2011). Age-dependent susceptibility of chromosome cohesion to premature separase activation in mouse oocytes. Biol. Reprod..

[66] Thomas C., Wetherall B., Levasseur M.D., Harris R.J., Kerridge S.T., Higgins J.M.G., Davies O.R., Madgwick S. (2021). A prometaphase mechanism of securin destruction is essential for meiotic progression in mouse oocytes. Nat. Commun..

[67] Touati S.A., Cladiere D., Lister L.M., Leontiou I., Chambon J.P., Rattani A., Bottger F., Stemmann O., Nasmyth K., Herbert M. (2012). Cyclin A2 Is Required for Sister Chromatid Segregation, But Not Separase Control, in Mouse Oocyte Meiosis. Cell Rep..

[68] Lee J., Kitajima T.S., Tanno Y., Yoshida K., Morita T., Miyano T., Miyake M., Watanabe Y. (2008). Unified mode of centromeric protection by shugoshin in mammalian oocytes and somatic cells. Nat. Cell Biol..

[69] Ishiguro T., Tanaka K., Sakuno T., Watanabe Y. (2010). Shugoshin-PP2A counteracts casein-kinase-1-dependent cleavage of Rec8 by separase. Nat. Cell Biol..

[70] Katis V.L., Lipp J.J., Imre R., Bogdanova A., Okaz E., Habermann B., Mechtler K., Nasmyth K., Zachariae W. (2010). Rec8 phosphorylation by casein kinase 1 and Cdc7-Dbf4 kinase regulates cohesin cleavage by separase during meiosis. Dev. Cell.

[71] Rumpf C., Cipak L., Dudas A., Benko Z., Pozgajova M., Riedel C.G., Ammerer G., Mechtler K., Gregan J. (2010). Casein kinase 1 is required for efficient removal of Rec8 during meiosis I. Cell Cycle.

[72] Rogers E., Bishop J.D., Waddle J.A., Schumacher J.M., Lin R. (2002). The aurora kinase AIR-2 functions in the release of chromosome cohesion in Caenorhabditis elegans meiosis. J. Cell Biol..

[73] Ferrandiz N., Barroso C., Telecan O., Shao N., Kim H.M., Testori S., Faull P., Cutillas P., Snijders A.P., Colaiacovo M.P. (2018). Spatiotemporal regulation of Aurora B recruitment ensures release of cohesion during C. elegans oocyte meiosis. Nat. Commun..

[74] Nguyen A.L., Schindler K. (2017). Specialize and Divide (Twice): Functions of Three Aurora Kinase Homologs in Mammalian Oocyte Meiotic Maturation. Trends Genet..

[75] Arguello-Miranda O., Zagoriy I., Mengoli V., Rojas J., Jonak K., Oz T., Graf P., Zachariae W. (2017). Casein Kinase 1 Coordinates Cohesin Cleavage, Gametogenesis, and Exit from M Phase in Meiosis II. Dev. Cell.

[76] Jonak K., Zagoriy I., Oz T., Graf P., Rojas J., Mengoli V., Zachariae W. (2017). APC/C-Cdc20 mediates deprotection of centromeric cohesin at meiosis II in yeast. Cell Cycle.

[77] El Yakoubi W., Wassmann K. (2017). Meiotic Divisions: No Place for Gender Equality. Adv. Exp. Med. Biol..

[78] Gomez R., Valdeolmillos A., Parra M.T., Viera A., Carreiro C., Roncal F., Rufas J.S., Barbero J.L., Suja J.A. (2007). Mammalian SGO2 appears at the inner centromere domain and redistributes depending on tension across centromeres during meiosis II and mitosis. EMBO Rep..

[79] Gryaznova Y., Keating L., Touati S.A., Cladiere D., El Yakoubi W., Buffin E., Wassmann K. (2021). Kinetochore individualization in meiosis I is required for centromeric cohesin removal in meiosis II. EMBO J..

[80] Mengoli V., Jonak K., Lyzak O., Lamb M., Lister L.M., Lodge C., Rojas J., Zagoriy I., Herbert M., Zachariae W. (2021). Deprotection of centromeric cohesin at meiosis II requires APC/C activity but not kinetochore tension. EMBO J..

[81] El Yakoubi W., Buffin E., Cladiere D., Gryaznova Y., Berenguer I., Touati S.A., Gomez R., Suja J.A., van Deursen J.M., Wassmann K. (2017). Mps1 kinase-dependent Sgo2 centromere localisation mediates cohesin protection in mouse oocyte meiosis I. Nat. Commun..

[82] Touati S.A., Buffin E., Cladiere D., Hached K., Rachez C., van Deursen J.M., Wassmann K. (2015). Mouse oocytes depend on BubR1 for proper chromosome segregation but not for prophase I arrest. Nat. Commun..

[83] Kim J., Ishiguro K., Nambu A., Akiyoshi B., Yokobayashi S., Kagami A., Ishiguro T., Pendas A.M., Takeda N., Sakakibara Y. (2015). Meikin is a conserved regulator of meiosis-I-specific kinetochore function. Nature.

[84] Maier N.K., Ma J., Lampson M.A., Cheeseman I.M. (2021). Separase cleaves the kinetochore protein Meikin at the meiosis I/II transition. Dev. Cell.

